# Characterization of a non-pathogenic H5N1 influenza virus isolated from a migratory duck flying from Siberia in Hokkaido, Japan, in October 2009

**DOI:** 10.1186/1743-422X-8-65

**Published:** 2011-02-11

**Authors:** Naoki Yamamoto, Yoshihiro Sakoda, Masayuki Motoshima, Fumi Yoshino, Kosuke Soda, Masatoshi Okamatsu, Hiroshi Kida

**Affiliations:** 1 Department of Disease Control, Graduate School of Veterinary Medicine, Hokkaido University, Kita 18, Nishi 9, Kita-ku, Sapporo, Hokkaido 060-0818, Japan; 2 Research Center for Zoonosis Control, Hokkaido University, Kita 20, Nishi 10, Kita-ku, Sapporo, Hokkaido 001-0020, Japan; 3 Japan Science and Technology Agency (JST), 4-1-8 Honcho, Kawaguchi, Saitama, 332-0012, Japan

## Abstract

**Background:**

Infection with H5N1 highly pathogenic avian influenza viruses (HPAIVs) of domestic poultry and wild birds has spread to more than 60 countries in Eurasia and Africa. It is concerned that HPAIVs may be perpetuated in the lakes in Siberia where migratory water birds nest in summer. To monitor whether HPAIVs circulate in migratory water birds, intensive surveillance of avian influenza has been performed in Mongolia and Japan in autumn each year. Until 2008, there had not been any H5N1 viruses isolated from migratory water birds that flew from their nesting lakes in Siberia. In autumn 2009, A/mallard/Hokkaido/24/09 (H5N1) (Mal/Hok/24/09) was isolated from a fecal sample of a mallard (*Anas platyrhynchos*) that flew from Siberia to Hokkaido, Japan. The isolate was assessed for pathogenicity in chickens, domestic ducks, and quails and analyzed antigenically and phylogenetically.

**Results:**

No clinical signs were observed in chickens inoculated intravenously with Mal/Hok/24/09 (H5N1). There was no viral replication in chickens inoculated intranasally with the isolate. None of the domestic ducks and quails inoculated intranasally with the isolate showed any clinical signs. There were no multiple basic amino acid residues at the cleavage site of the hemagglutinin (HA) of the isolate. Each gene of Mal/Hok/24/09 (H5N1) is phylogenetically closely related to that of influenza viruses isolated from migratory water birds that flew from their nesting lakes in autumn. Additionally, the antigenicity of the HA of the isolate was similar to that of the viruses isolated from migratory water birds in Hokkaido that flew from their northern territory in autumn and different from those of HPAIVs isolated from birds found dead in China, Mongolia, and Japan on the way back to their northern territory in spring.

**Conclusion:**

Mal/Hok/24/09 (H5N1) is a non-pathogenic avian influenza virus for chickens, domestic ducks, and quails, and is antigenically and genetically distinct from the H5N1 HPAIVs prevailing in birds in Eurasia and Africa. H5 viruses with the HA gene of HPAIV had not been isolated from migratory water birds in the surveillance until 2009, indicating that H5N1 HPAIVs had not become dominant in their nesting lakes in Siberia until 2009.

## Background

Influenza viruses widely distribute in birds and mammals including humans. Viruses of each of the known hemagglutinin (HA) and neuraminidase (NA) subtypes (H1-H16 and N1-N9, respectively) have been isolated from migratory water birds. Ducks are orally infected with influenza viruses by waterborne transmission at their nesting lakes in Siberia, Alaska, and Canada close to the Arctic Circle during their breeding season, in summer [1]. These viruses replicate in the columnar epithelial cells forming crypts in the colon, and are excreted in feces [2]. The viruses are preserved in frozen lake water in winter after the ducks leave for migration to the south [3]. Nesting lakes for migratory ducks, thus, serve as influenza virus gene pools in nature.

Since late 2003, H5N1 highly pathogenic avian influenza viruses (HPAIVs) have seriously affected poultry in Eurasia and Africa. Non-pathogenic avian influenza viruses (NPAIVs) circulating in waterfowl transmit to terrestrial birds such as quails and turkeys through domestic water birds such as ducks and geese in live bird markets. Then HPAIVs are generated during multiple transmission of low pathogenic H5 or H7 viruses in chicken population [1]. After 2005, H5N1 HPAIVs have been isolated from dead migratory water birds in China, Mongolia, Russia, and Japan on the way back to their nesting lakes in Siberia in spring [4-8]. It is a serious concern that HPAIVs may be perpetuated in the lakes where migratory water birds nest in summer, and that those migratory water birds may then bring HPAIVs to the south in autumn.

Since Japan and Mongolia are located on the flyways of migratory water birds that flew from their nesting lakes in Siberia to the south [1,9-11], intensive surveillance of avian influenza has been performed in autumn in Hokkaido, Japan, and Mongolia every year since 1996. The subtypes and the numbers of isolates in the surveillance in autumn between 1996 and 2009 have been reported [6,11-13]. A total of 634 viruses including 17 H5 viruses were isolated from fecal samples of migratory water birds in the surveillance (Tables 1 and 2). Until 2008, H5N1 virus had not been isolated from those of migratory water birds. In autumn 2009, an H5N1 virus, A/mallard/Hokkaido/24/09 (H5N1) (Mal/Hok/24/09), was isolated from the fecal sample of a mallard (*Anas platyrhynchos*) in Hokkaido, Japan. Pathogenicity of the isolate for chickens, domestic ducks, and quails was assessed by experimental infection studies, and the isolate was phylogenetically and antigenically analyzed.

**Table 1:** Influenza viruses isolated from fecal samples of migratory water birds in autumn between 1996 and 2009

Locations	Subtypes of influenza viruses isolated in following years													
	
	1996	1997	1998	1999	2000	2001	2002	2003	2004	2005	2006	2007	2008	2009
Sapporo,	NP^a^	NP	NP	NP	NP	H1N1 (9)	H3N8 (3)	H3N8 (11)	H1N1 (1)	H3N2 (1)	H3N8 (5)	H3N8 (2)	H3N2 (1)	H1N3 (1)
Japan						H3N6 (1)	H5N3 (1)	H6N8 (2)	H3N8 (1)	H6N2 (4)	H4N6 (1)	H4N6 (2)	H3N6 (3)	H1N5 (1)
						H4N5 (1)	H11N9 (3)	H7N1 (18)	H4N2 (7)	H8N4 (2)	H6N2 (1)	H5N3 (2)	H4N6 (9)	H4N6 (5)
						H4N6 (1)		H8N4 (1)	H5N3 (3)		H9N2 (1)	H8N4 (2)	H7N7 (1)	H5N1 (1)
									H6N1 (5)		H11N9 (3)	H12N5 (1)	H9N5 (1)	H6N1 (4)
									H6N8 (2)				H10N7 (11)	H6N8 (2)
									H10N5 (7)					H11N9 (3)
														H12N5 (1)
														
Wakkanai,	H1N1 (1)^b^	H1N1 (1)	H6N2 (1)	H2N2 (1)	H4N6 (1)	H2N2 (1)	NP	NP	H4N6 (6)	H2N5 (1)	H3N6 (2)	H1N1 (1)	H4N6 (2)	H5N2 (1)
Japan	H3N8 (1)	H6N1 (2)	H9N2 (1)	H3N8 (2)	H5N3 (2)	H2N3 (4)			H6N2 (12)	H3N8 (3)	H3N8 (1)	H3N8 (1)	H5N2 (1)	
	H5N3 (3)	H9N2 (1)		H6N2 (4)	H6N2 (2)	H3N8 (6)			H6N8 (2)	H6N1 (1)	H4N9 (3)	H4N6 (2)	H6N1 (4)	
	H6N1 (1)	H11N9 (1)		H9N2 (2)	H8N4 (1)	H6N2 (4)			H7N7 (13)	H6N2 (3)	H6N1 (4)	H8N4 (1)	H6N2 (1)	
					H9N2 (1)	H12N5 (2)			H8N4 (1)		H6N5 (1)	H10N2 (1)	H6N5 (1)	
					H10N4 (12)				H10N6 (1)		H9N2 (1)	H10N7 (1)	H6N8 (1)	
									H11N9 (1)		H10N8 (1)		H6N9 (1)	
									H12N5 (1)		H11N9 (11)		H9N9 (1)	
											H13N6 (2)		H10N9 (2)	
													H11N9 (2)	
														
Mongolia	NP	NP	NP	NP	NP	H1N1 (1)	H1N1 (3)	H1N1 (1)	NP	H3N2 (1)	H2N2 (1)	H3N8 (14)	H3N6 (3)	H1N8 (1)
						H3N2 (1)	H3N6 (20)	H2N3 (1)		H3N6 (2)	H3N8 (8)	H4N3 (1)	H3N8 (23)	H3N8 (2)
						H3N6 (3)	H3N8 (55)	H3N6 (6)		H3N8 (10)	H4N6 (9)	H7N6 (1)	H4N6 (8)	H4N6 (3)
						H3N8 (11)	H4N6 (12)	H3N8 (28)		H4N6 (6)		H7N7 (4)	H4N8 (3)	H8N4 (3)
						H4N2 (1)	H4N7 (1)	H4N2 (1)		H8N4 (1)			H7N9 (3)	
						H4N6 (12)	H4N8 (1)	H4N6 (25)		H10N3 (11)				
						H5N2 (1)	H7N1 (1)	H9N2 (1)		H10N7 (1)				
						H5N3 (2)	H7N7 (9)	H10N5 (5)						
						H7N1 (1)	H8N4 (5)							
						H10N3 (4)	H10N7 (1)							
							H12N5 (1)							

Surveillance data were referred from Okazaki *et al. *[11], Manzoor *et al. *[12], Sakoda *et al. *[6], and Asmah *et al*.[13].

^a ^Surveillance did not be performed.

^b ^Number of isolates of each antigenic subtype is shown in parenthesis.

**Table 2:** H5 viruses isolated from migratory water birds in the surveillance in autumn between 1996 and 2009

Years	Locations	Names	Subtypes
1996	Wakkanai, Japan	Swan/Hokkaido/4/96	H5N3
		Swan/Hokkaido/51/96	H5N3
		Swan/Hokkaido/67/96	H5N3
2000	Wakkanai, Japan	Dk/Hokkaido/447/00	H5N3
		Dk/Hokkaido/69/00	H5N3
2001	Mongolia	Dk/Mongolia/54/01	H5N2
		Dk/Mongolia/500/01	H5N3
		Dk/Mongolia/596/01	H5N3
2002	Sapporo, Japan	Dk/Hokkaido/84/02	H5N3
2004	Sapporo, Japan	Dk/Hokkaido/101/04	H5N3
		Dk/Hokkaido/193/04	H5N3
		Dk/Hokkaido/299/04	H5N3
2007	Sapporo, Japan	Dk/Hokkaido/167/07	H5N3
		Dk/Hokkaido/201/07	H5N3
2008	Wakkanai, Japan	Dk/Hokkaido/WZ21/08	H5N2
2009	Wakkanai, Japan	Dk/Hokkaido/W75/09	H5N2
	Sapporo, Japan	Mal/Hokkaido/24/09	H5N1

Abbreviations: Dk (Duck), Mal (Mallard).

## Materials and methods

### Isolation and identification of viruses

A total of 711 fecal samples were collected from migratory water birds at lakeside of Ono Pond on the campus of Hokkaido University, Sapporo and Lake Ohnuma in Wakkanai, Hokkaido, Japan, between September and November 2009. Each sample was mixed with Minimum Essential Medium (Nissui) containing antibiotics and inoculated into the allantoic cavities of ten-day-old chicken embryos. The subtypes of influenza viruses were identified by hemagglutination inhibition (HI) and neuraminidase inhibition (NI) tests with antisera to the reference influenza virus strains[14].

### Sequencing and phylogenetic analysis

Viral RNA was extracted from the allantoic fluid of chicken embryos infected with the isolates by TRIzol LS Reagent (Invitrogen) and reverse-transcribed with the Uni12 primer [15] and SuperScript Reverse Transcriptase III (Invitrogen) or M-MLV Reverse Transcriptase (Invitrogen). The full-length of each gene segment was amplified by polymerase chain reaction with gene-specific primer sets [15]. Direct sequencing of each gene segment was performed using an auto-sequencer CEQ 2000XL (Beckman Coulter) or 3500 Genetic Analyzer (Applied Biosystems).

The nucleotide sequences were phylogenically analyzed based on those of the H5 HA and N1 NA genes of influenza viruses by the neighbor-joining method [3,16]. Sequence data of the viral genes were compared with those from GenBank/EMBL/DDBJ.

### Experimental infection of chickens, domestic ducks, and quails with Mal/Hok/24/09 (H5N1)

To determine the intravenous pathogenicity index (IVPI), 0.2 ml of the 1:10 dilution of infectious allantoic fluid of embryonated eggs was inoculated intravenously into ten seven-week-old chickens (White Leghorn). The score for IVPI was calculated according to the manual of World Organisation for Animal Health (OIE) [17].

To assess the intranasal pathogenicity for poultry, Mal/Hok/24/09 (H5N1) of 10^6.0 ^50% egg infectious dose (EID_50_) was inoculated intranasally into eight four-week-old chickens (Boris Brown), domestic ducks (Chelly Valley), and quails (Japanese Quail). Four of eight birds were euthanized three days post-inoculation (dpi), and the trachea and cloaca swabs, brain, trachea, lungs, kidneys, and colon were collected aseptically for virus recovery. The birds were observed daily for disease signs for 14 days after inoculation. Sera were collected from them on the day of inoculation and 14 dpi to test for antibodies against H5N1 virus. The swabs and tissue homogenates were inoculated into ten-day-old chicken embryos and the infectivity titers of virus were calculated and expressed as the EID_50 _per milliliter of swab or gram of tissue samples. Sera were examined for the presence of antibodies against H5N1 virus by enzyme-linked immunosorbent assay (ELISA) [18]. The purified A/duck/Hokkaido/Vac-1/04 (H5N1) generated from H5N2 and H7N1 viruses isolated from migratory water birds by genetic reassortment in embryonated chicken eggs [19] was used as antigen for ELISA. Each of the birds was housed in a self-contained isolator unit (Tokiwa Kagaku) at a BSL-3 facility at the Graduate School of Veterinary Medicine, Hokkaido University, Japan.

### Antigenic analysis

The antigenic properties of H5 viruses, A/duck/Hokkaido/WZ21/08 (H5N2), A/duck/Hokkaido/WZ75/09 (H5N2), Mal/Hok/24/09 (H5N1), A/whooper swan/Hokkaido/1/08 (H5N1), and A/peregrine falcon/Hong Kong/810/09 (H5N1), were determined by the fluorescent antibody method with monoclonal antibodies (MAbs) against H5 HA produced previously [20]. MDCK cells infected with H5 influenza viruses were fixed with cold 100% acetone for eight hours post-inoculation. The reactivity patterns of the MAbs to H5 viruses were investigated by the immunofluorescent method with a FITC-conjugated goat IgG to mouse IgG (ICN Biomedicals). Fluorescence was visualized with the Axiovert 200 (Carl Zeiss).

## Results

### Isolation of influenza A viruses from fecal samples of migratory water birds

In 2009, a total of 19 viruses were isolated from 711 fecal samples of migratory water birds. Those were 1 H1N3, 1 H1N5, 5 H4N6, 1 H5N1, 1 H5N2, 4 H6N1, 2 H6N8, 3 H11N9, and 1 H12N5 viruses. In our previous surveillance until 2008, H5N1 virus had not been isolated (Table 1) [6,11-13]. In autumn 2009, Mal/Hok/24/09 (H5N1) was isolated from a fecal sample of a mallard in Hokkaido, Japan.

### Pathogenicity of Mal/Hok/24/09 (H5N1) in chickens, domestic ducks, and quails

The pathogenicity of Mal/Hok/24/09 (H5N1) was evaluated by IVPI test using chickens. None of the ten birds intravenously inoculated with Mal/Hok/24/09 (H5N1) showed clinical signs during ten days of observation (IVPI = 0.00). None of the chickens, domestic ducks, and quails intranasally inoculated with 10^6.0 ^EID_50 _of Mal/Hok/24/09 (H5N1) showed clinical signs during 14 days of observation (Table 3). The virus was not recovered from the tracheal and cloacal swabs and tissues of chickens intranasally inoculated with Mal/Hok/24/09 (H5N1) on three dpi, and there were no antibodies to H5N1 virus detected by ELISA on 14 dpi (Table 3), indicating that chickens were not infected with the isolate. Although virus was not recovered from the swabs and tissues of domestic ducks inoculated with the virus on three dpi, antibodies against H5N1 virus were detected in the sera of the birds, indicating that domestic ducks were infected with the isolate. Viruses of 10^3.3 ^and 10^3.6 ^EID_50_/ml were recovered from tracheal swabs of two of four quails inoculated with the virus on three dpi, respectively. Antibodies against H5N1 virus were detected in the sera of the birds on 14 dpi. These findings indicate that quails are susceptible to infection with the isolate.

**Table 3:** Virus recovery from birds experimentally inoculated with A/mallard/Hokkaido/24/09 (H5N1)

				Virus recovery^d^							
					
Birds	No. of Birds	Days^a^ **p.i**.	Clinical signs	Swabs (log EID_50_/ml)		Tissues (log EID_50_/g)					Antibody^e ^response
						
				Trachea	Cloaca	Brain	Trachea	Lungs	Kidneys	Colon	
Chickens	1 - 4	3	-^b^	<	<	<	<	<	<	<	NT
	5 - 8	14	-	NT^c^	NT	NT	NT	NT	NT	NT	< 40^f^
											
Domestic ducks	1 - 4	3	-	<	<	<	<	<	<	<	NT
	5 - 8	14	-	NT	NT	NT	NT	NT	NT	NT	1,600^f^
											
Quails	1	3	-	3.3	<	<	<	<	<	<	NT
	2	3	-	3.6	<	<	<	<	<	<	NT
	3 - 4	3	-	<	<	<	<	<	<	<	NT
	5 - 8	14	-	NT	NT	NT	NT	NT	NT	NT	800^f^

^a ^All birds were sacrificed.

^b ^-: Birds did not show any clinical signs during observation days.

^c ^NT: Not tested.

^d ^Digit: Virus titers. <: Virus titer was less than 0.8 log EID_50_/ml swab or 1.5 log EID_50_/g tissue.

^e ^ELISA titers on 14 dpi.

^f ^ELISA titers of three birds were equal.

### Genetic analysis of Mal/Hok/24/09 (H5N1)

**Figure 1:**
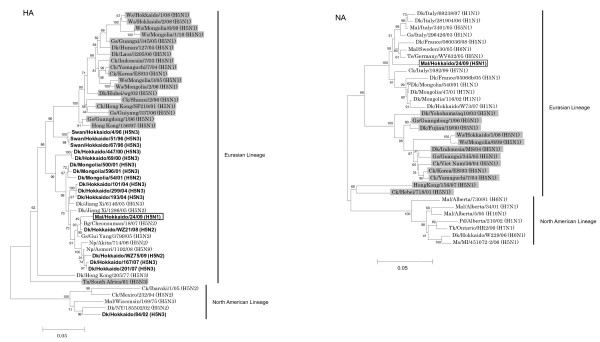
**Phylogenetic trees of the H5 HA and N1 NA genes of influenza viruses**. Nucleotides 79 - 1,024 (946 bp) of the HA and 226 - 1,098 (873 bp) of the NA were used for phylogenetic analysis. Horizontal distances are proportional to the minimum number of nucleotide differences required to join nodes and sequences. Digits at the nodes indicate the probability of confidence levels in a bootstrap analysis with 1,000 replications. HPAIVs are highlighted in gray. Mal/Hok/24/09 (H5N1) is shown in the enclosed square. H5 viruses isolated from migratory water birds in the surveillance in autumn between 1996 and 2009 are denoted in bold. Abbreviations: Ws (Whooper swan), Gs (Goose), Dk (Duck), Ck (Chicken), Mal (Mallard), Bg (Bean goose), Np (Northern pintail), Tn (Tern), Gu (Gull), Te (Teal), Ms (Mute swan), Pd (Pintail duck), Tk (Turkey).

Each gene of Mal/Hok/24/09 (H5N1) was phylogenetically analyzed. The HA and NA genes of Mal/Hok/24/09 (H5N1) were classified into the Eurasian lineage, and were different from HA and NA genes of H5N1 HPAIVs, respectively (Figure 1). In addition, the other six genes of Mal/Hok/24/09 (H5N1) were not closely related to those of HPAIVs, but related to those of NPAIVs isolated from migratory water birds (data not shown). The eight segments of Mal/Hok/24/09 (H5N1) were analyzed by the Basic Local Alignment Search Tool (BLAST) available from the DDBJ/EMBL/GenBank (Table 4). It was found that all genes of Mal/Hok/24/09 (H5N1) were derived from those of the viruses circulating in water birds in nature. M gene of the virus was classified into North American lineage, and the other genes were classified into Eurasian lineage (Table 4), indicating that genetic reassortment occurs between the viruses whose genes classified into North American and Eurasian lineages. The amino acid sequence of the HA cleavage site of Mal/Hok/24/09 (H5N1) was RETR/GLF, and insertion or substitution of multiple basic amino acids found in the HAs of HPAIVs [21] was not observed.

**Table 4:** Characterization of the genes of A/mallard/Hokkaido/24/09 (H5N1)

		Viruses with highest homology		Homologies (%)	Lineages
		
Gene segments^a^	Region of examined nucleotides	Name^b^	Accession numbers		
PB2	14-2293	Sbd/Korea/619/08 (H6N2)	GQ414790	98	Eurasian
PB1	9-2269	Sbd/Korea/540/08 (H6N1)	GQ414822	98	Eurasian
PA	1-2200	Dk/Shiga/8/04 (H4N6)	AB304146	98	Eurasian
HA	79-1726	Bg/Cheonsuman/18/07 (H5N2)	FJ767718	98	Eurasian
NP	31-1527	Mal/SanJiang/151/06 (H6N2)	EF592496	99	Eurasian
NA	1-1422	Gs/Italy/296426/03 (H1N1)	FJ432780	97	Eurasian
M	1-983	Mal/Minnesota/153/98 (H9N2)	GU051519	98	North American
NS	1-838	Gu/Astrakhan/1846/98 (H13N6)	GU052231	98	Eurasian

^a ^GenBank accession number of each gene of Mal/Hokkaido/24/09 (H5N1): PB2 [AB530989], PB1 [AB530990], PA [AB530991], HA [AB530992],

NP [AB530993], NA [AB530994], M [AB530995], and NS [AB530996].

^b ^Abbreviations: Sbd (Spot-billed duck), Dk (Duck), Bg (Bean goose), Mal (Mallard), Gs (Goose), Gu (Gull).

### Antigenic analysis of the HA of Mal/Hok/24/09 (H5N1)

The HA of Mal/Hok/24/09 (H5N1) was antigenically analyzed using a panel of MAbs recognizing six different epitopes on the HA of A/duck/Pennsylvania/10218/84 (H5N2) [20]. Each of the MAb bound to the antigen of Mal/Hok/24/09 (H5N1) as well as those of the other non-pathogenic H5 viruses, and few MAbs bound to the antigen of H5N1 HPAIVs recently isolated in Mongolia, Japan, and Hong Kong (Table 5), indicating that the HA of Mal/Hok/24/09 (H5N1) is antigenically closely related to the H5 HA of the viruses circulating in migratory water bird.

**Table 5:** Reactivity of H5 viruses with MAbs against HA of A/duck/Pennsylvania/10218/84 (H5N2)

			Monoclonal antibodies					
			
Viruses^a^		Clades	D101/1 (88^b^)	A310/39 (145)	64/1 (157)	B9/5 (168)	B59/5 (169)	25/2 (205)
NPAIVs	Dk/Pennsylvania/10218/84 (H5N2)	-^c^	+	+	+	+	+	+
	Swan/Hokkaido/4/96 (H5N3)	-	+	+	+	+	+	+
	Swan/Hokkaido/51/96 (H5N3)	-	+	+	+	+	+	+
	Swan/Hokkaido/67/96 (H5N3)	-	+	+	+	+	+	+
	Dk/Hokkaido/447/00 (H5N3)	-	+	+	+	+	+	+
	Dk/Hokkaido/69/00 (H5N3)	-	+	+	+	+	+	+
	Dk/Mongolia/54/01 (H5N2)	-	+	+	+	+	+	+
	Dk/Mongolia/500/01 (H5N3)	-	+	+	+	+	+	+
	Dk/Mongolia/596/01 (H5N3)	-	+	+	+	+	+	+
	Dk/Hokkaido/84/02 (H5N3)	-	+	+	+	+	+	+
	Dk/Hokkaido/101/04 (H5N3)	-	+	+	+	+	+	+
	Dk/Hokkaido/193/04 (H5N3)	-	+	+	+	+	+	+
	Dk/Hokkaido/299/04 (H5N3)	-	+	+	+	+	+	+
	Dk/Hokkaido/167/07 (H5N3)	-	+	+	+	+	+	+
	Dk/Hokkaido/201/07 (H5N3)	-	+	+	+	+	+	+
	Dk/Hokkaido/WZ21/08 (H5N2)	-	+	+	+	+	+	+
	Dk/Hokkaido/WZ75/09 (H5N2)	-	+	+	+	+	+	+
	Mal/Hokkaido/24/09 (H5N1)	-	+	+	+	+	+	+
								
HPAIVs	Ws/Mongolia/3/05 (H5N1)	2.2	+	-	+	+	-	+
	Ws/Hokkaido/1/08 (H5N1)	2.3.2	+	-	-	-	-	-
	Pf/Hong Kong/810/09 (H5N1)	2.3.4	-	-	-	-	-	-

The results, except Dk/Hokkaido/WZ21/08 (H5N2), Dk/Hokkaido/WZ75/09 (H5N2), Mal/Hokkaido/24/09 (H5N1), Ws/Hokkaido/1/08 (H5N1), and Pf/Hong Kong/810/09 (H5N1), were referred from previous report [20].

^a ^Abbreviations: Dk (Duck), Mal (Mallard), Ws (Whooper swan), Pf (Peregrine falcon).

^b ^Location of amino acid substitutions in antigenic variants selected in the presence of respective monoclonal antibodies [20].

^c ^Dashes (-) indicate classical HA gene which is not classified into the clades 0 - 9.

## Discussion

Efforts to monitor avian influenza in migratory water birds have increased worldwide in recent years due to concern that migratory water birds may disseminate HPAIVs. Intensive surveillance of avian influenza has been conducted every autumn in Hokkaido, Japan, and Mongolia. As shown in Table 1, H5N1 virus had not been isolated from migratory water birds in the surveillance until 2008. In autumn 2009, Mal/Hok/24/09 (H5N1) was isolated from a fecal sample of a mallard that flew from the northern territory in Siberia to Hokkaido, Japan. In the present study, the H5N1 isolate was examined for pathogenicity in chickens, domestic ducks, and quails. Based on the results of IVPI test, the isolate was designated a NPAIV. Chickens were not susceptible to infection with Mal/Hok/24/09 (H5N1) (Table 3). Domestic ducks and quails were infected with the isolate but did not show clinical signs. These findings indicate that the isolate is non-pathogenic in chickens, domestic ducks, and quails. Phylogenetic analyses demonstrated that Mal/Hok/24/09 (H5N1) was distinguished from H5N1 HPAIVs that are prevailing in birds in Eurasia and Africa. Antigenic comparisons of the HAs of H5 viruses indicated that the antigenicity of the HA of Mal/Hok/24/09 (H5N1) is closely related with the H5 NPAIVs circulating in nature (Table 5).

After 1996, H5N1 HPAIVs with both HA and NA genes of A/goose/Guangdong/1/96 (H5N1) have spread to Eurasia and Africa [22]. After 2005, H5N1 HPAIVs were isolated from dead migratory water birds in China, Mongolia, Russia, and Japan in spring [4-8], suggesting that the birds were infected with HPAIVs in the south during the spring and died on the way back to the northern territories. In the surveillance studies of avian influenza in autumn since 1996, H5 viruses with the HA gene of A/goose/Guangdong/1/96 (H5N1) had not been isolated from migratory water birds that flew from Siberia to Japan and Mongolia (Figure 1) indicating that H5N1 HPAIVs had not become dominant in their nesting lakes in Siberia until 2009.

On 14^th ^October, 2010, H5N1 HPAIVs were isolated from migratory water birds that flew from Siberia to Japan (under publication). Then, H5N1 HPAIVs have been isolated from migratory water birds and poultry in other places in Japan.

For the control of HPAIV infection in birds and mammals, early detection of the viruses and stamping out to contain the viruses in the domestic poultry are essential.

## Conclusion

In autumn 2009, Mal/Hok/24/09 (H5N1) was isolated from a fecal sample of a mallard. Mal/Hok/24/09 (H5N1) is a NPAIV for chickens, domestic ducks, and quails, and is antigenically and genetically distinct from H5N1 HPAIVs that are prevailing in birds in Eurasia and Africa. Phylogenetic analysis of the HA genes revealed that H5 viruses with the HA gene of HPAIV had not been isolated from migratory water birds in the surveillance until 2009. These findings indicate that H5N1 HPAIVs had not become dominant in their nesting lakes in Siberia until 2009.

## Competing interests

The authors declare that they have no competing interests.

## Authors' contributions

NY carried out the animal experiments and the antigenic and phylogenetic analyses, and drafted the manuscript. MM and FY collected the fecal samples and carried out the viral isolation and identification of subtypes. MO carried out the IVPI test. KS participated in the antigenic analysis. YS and HK participated in coordination of the study. All authors read and approved the final manuscript.
